# AI in Dental Radiology—Improving the Efficiency of Reporting With ChatGPT: Comparative Study

**DOI:** 10.2196/60684

**Published:** 2024-12-23

**Authors:** Daniel Stephan, Annika Bertsch, Matthias Burwinkel, Shankeeth Vinayahalingam, Bilal Al-Nawas, Peer W Kämmerer, Daniel GE Thiem

**Affiliations:** 1 Department of Oral and Maxillofacial Surgery, Facial Plastic Surgery University Medical Centre of the Johannes Gutenberg-University Mainz Mainz Germany; 2 Department of Oral and Maxillofacial Surgery Radboud University Medical Center Nijmegen Netherlands

**Keywords:** artificial intelligence, ChatGPT, radiology report, dental radiology, dental orthopantomogram, panoramic radiograph, dental, radiology, chatbot, medical documentation, medical application, imaging, disease detection, clinical decision support, natural language processing, medical licensing, dentistry, patient care

## Abstract

**Background:**

Structured and standardized documentation is critical for accurately recording diagnostic findings, treatment plans, and patient progress in health care. Manual documentation can be labor-intensive and error-prone, especially under time constraints, prompting interest in the potential of artificial intelligence (AI) to automate and optimize these processes, particularly in medical documentation.

**Objective:**

This study aimed to assess the effectiveness of ChatGPT (OpenAI) in generating radiology reports from dental panoramic radiographs, comparing the performance of AI-generated reports with those manually created by dental students.

**Methods:**

A total of 100 dental students were tasked with analyzing panoramic radiographs and generating radiology reports manually or assisted by ChatGPT using a standardized prompt derived from a diagnostic checklist.

**Results:**

Reports generated by ChatGPT showed a high degree of textual similarity to reference reports; however, they often lacked critical diagnostic information typically included in reports authored by students. Despite this, the AI-generated reports were consistent in being error-free and matched the readability of student-generated reports.

**Conclusions:**

The findings from this study suggest that ChatGPT has considerable potential for generating radiology reports, although it currently faces challenges in accuracy and reliability. This underscores the need for further refinement in the AI’s prompt design and the development of robust validation mechanisms to enhance its use in clinical settings.

## Introduction

Structured and standardized documentation plays a crucial role in health care by ensuring accurate recording and communication of diagnostic findings, treatment plans, and patient progress, thereby supporting high-quality patient care [1]. However, manual documentation is often time-consuming, error-prone, and can impede clinical workflow efficiency, especially in fast-paced medical settings. With the emergence of artificial intelligence (AI), there is a growing interest in implementing AI technology to optimize health care workflows and improve documentation practices.

AI has proven useful in various medical applications, from diagnosing diseases to drug development [2]. In radiology, AI algorithms analyze medical images to assist in early disease detection, improve radiologists’ performance, and provide clinical decision support [3,4]. Moreover, AI-driven solutions have the potential to automate repetitive tasks and reduce the workload of health care professionals [5,6].

First introduced by OpenAI in 2018, (GPT—a specific large language model developed by OpenAI) has continuously evolved and trained on extensive text data [7]. ChatGPT (implementation of GPT), an advanced large language model (a class of AI models), represents a significant advancement in natural language processing and has demonstrated remarkable capabilities in understanding and generating human-like text using deep learning techniques, like neuronal networks. GPT 3.5 showed a human-level performance across various medical exams and passed the United States Medical Licensing Exam (60.2%), Med-MCQA (57.5%), and PubMedQA (78.2%) [8-10]. With its proficiency in language generation, ChatGPT is capable of medical writing [11] and, therefore, has been increasingly integrated into medical education [12] and clinical practice, allowing it to automate the writing of examination findings, doctor’s letters, or radiology reports [13].

Dental radiology, integral to dentistry, relies on the correct interpretation of x-ray images, including panoramic radiographs (OPG), to diagnose and plan numerous oral conditions or pathologies. To maintain the standard of patient care, it is, therefore, crucial to ensure high-quality training in radiology tasks during dental studies. Traditionally, radiology education involves manual interpretation of x-ray images and writing detailed medical findings reports based on visual inspection and clinical knowledge. However, the emergence of AI technologies has increased interest in alternative methods for radiology education and diagnostic reporting, including maxillofacial radiology [14,15].

The capability of AI in diagnosing medical images, including x-ray images, is well-established [3,16]. Moreover, studies have demonstrated that ChatGPT can generate clinic letters and operative notes with high correctness and readability [17,18]. Additionally, another study has proven its efficacy beyond text generation in simplifying existing radiology reports and improving patient understanding [19]. Furthermore, recent research reveals AI’s capability to outperform dental students in diagnostic accuracy regarding endodontic assessments [20], highlighting its potential as a reference tool to enhance students’ understanding and diagnostic skills. However, this raises concerns about the potential for overreliance on AI, considering reports about ChatGPT generating fake findings for imaginable diseases [21], which may affect the development of critical analytical and decision-making abilities. Thus, it is essential to integrate AI with human expertise and clinical judgment in dental education. ChatGPT shows promising potential in improving doctor-patient communication by simplifying complex medical information and transforming complex medical terminology into easily understandable language for patients with varying levels of health expertise [22]. While earlier versions of ChatGPT powered by GPT-3.5 generated patient-facing information lacking accuracy and important information, GPT-4 has shown improvements in appropriateness and accuracy and, despite occasional omissions, ultimately produced patient information applicable for gaining informed consent for procedures in nuclear medicine [23].

Nevertheless, the generation of radiology reports based on diagnostic findings by health care professionals remains a subject of investigation. Therefore, this study evaluated the efficacy of incorporating AI language models, specifically ChatGPT, into generating radiology reports. Dental students analyzed OPGs and provided diagnoses through checkbox lists together with written reports. A comparative analysis between radiology reports manually written by dental students and reports generated by the AI based on those prefilled checkbox lists was conducted. This study primarily investigated the readability of both report types with the null hypothesis stating no differences in readability between the 2 sets of reports. Secondary outcomes, including text accuracy and language quality, were evaluated to identify potential areas for improvement in AI-driven radiology reporting.

## Methods

### Overview

This study sought to investigate the efficacy of incorporating AI language models, specifically ChatGPT, in generating radiology reports from prefilled checkbox lists after analyzing OPGs. Dental students were assigned to diagnose 2 different x-ray images, providing a written radiology report for 1 and a checkbox list of diagnoses for the other. The AI then generated reports based on the diagnoses provided within the checkbox lists. Subsequently, both texts were analyzed comparatively to primarily evaluate readability, with a secondary evaluation of text quality, accuracy, similarities, and disparities between student-written and AI-written reports.

### Ethical Considerations

The study adhered strictly to ethical standards and institutional guidelines, obtaining informed consent from all participants beforehand. Participants were clearly informed of the purpose of the research, the voluntary nature of participation, and their ability to withdraw at any time without any repercussions. Additionally, no compensation was provided, as the tasks were integral to students’ academic training. Confidentiality and data privacy were stringently maintained throughout the research process to uphold the participant’s well-being and privacy. An ethics approval was not required as the generation of radiology reports is a standard component of dental education in Germany and this type of research does not involve intervention or data collection beyond routine educational activities. The tasks performed by the students were part of their regular academic curriculum and no additional tasks outside the students’ regular academic curriculum were introduced. Moreover, the analysis of data was conducted anonymously, ensuring privacy and confidentiality and preventing participating in this study resulted in either advantages or disadvantages for the students. As no images or materials involving identifiable features were included in this study, no additional consent forms were required.

### Study Setting

The study took place in the radiology section of the Department of Oral and Maxillofacial Surgery at the University Medical Centre Mainz, Germany. Certified medical monitors were provided, and all participants were supervised throughout the session without access to additional information or external help.

### Participants

In Germany, dental education is structured into 10 semesters. The first 5 semesters focus on foundational knowledge, while the following 5 semesters (clinical semesters 1-5, corresponding to overall semesters 6-10) emphasize clinical skills. The first lesson in dental radiology was introduced in the first clinical semester and, therefore, preclinical students were excluded from this study. A total of 100 dental students from all 5 clinical semesters participated in the study with the following distribution across semesters—semester 1: n=20, semester 2: n=19, semester 3: n=21, semester 4: n=20, and semester 5: n=20. This equal representation across different stages of dental education highlights the progressive development in radiology report writing.

### Experimental Design

**Figure 1:**
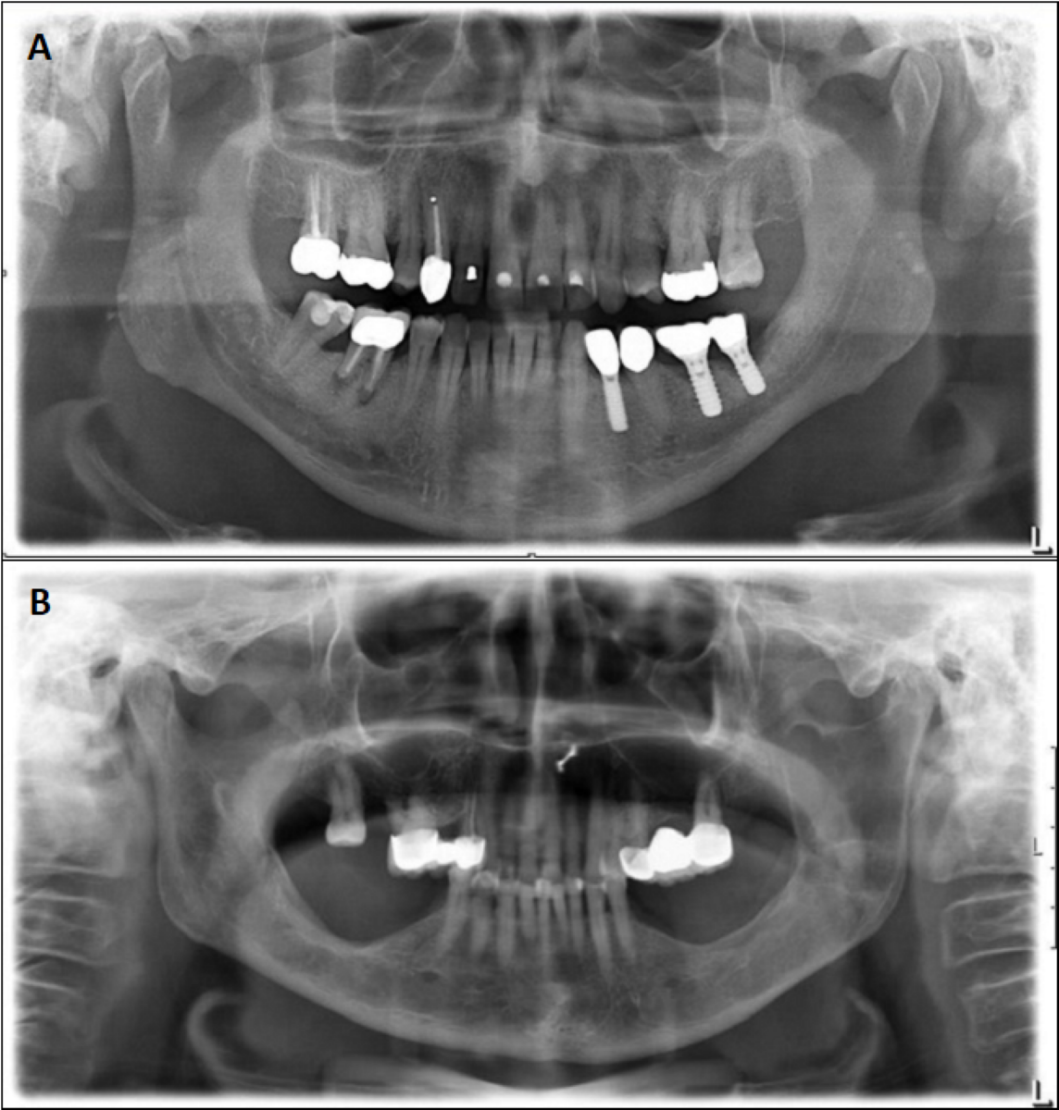
(A,B) Two randomly chosen panoramic radiographs featuring various pathologies to be diagnosed by dental students. Both x-ray images represented the basis of a student-written and an AI-generated radiology report. AI: artificial intelligence.

**Figure 2:**
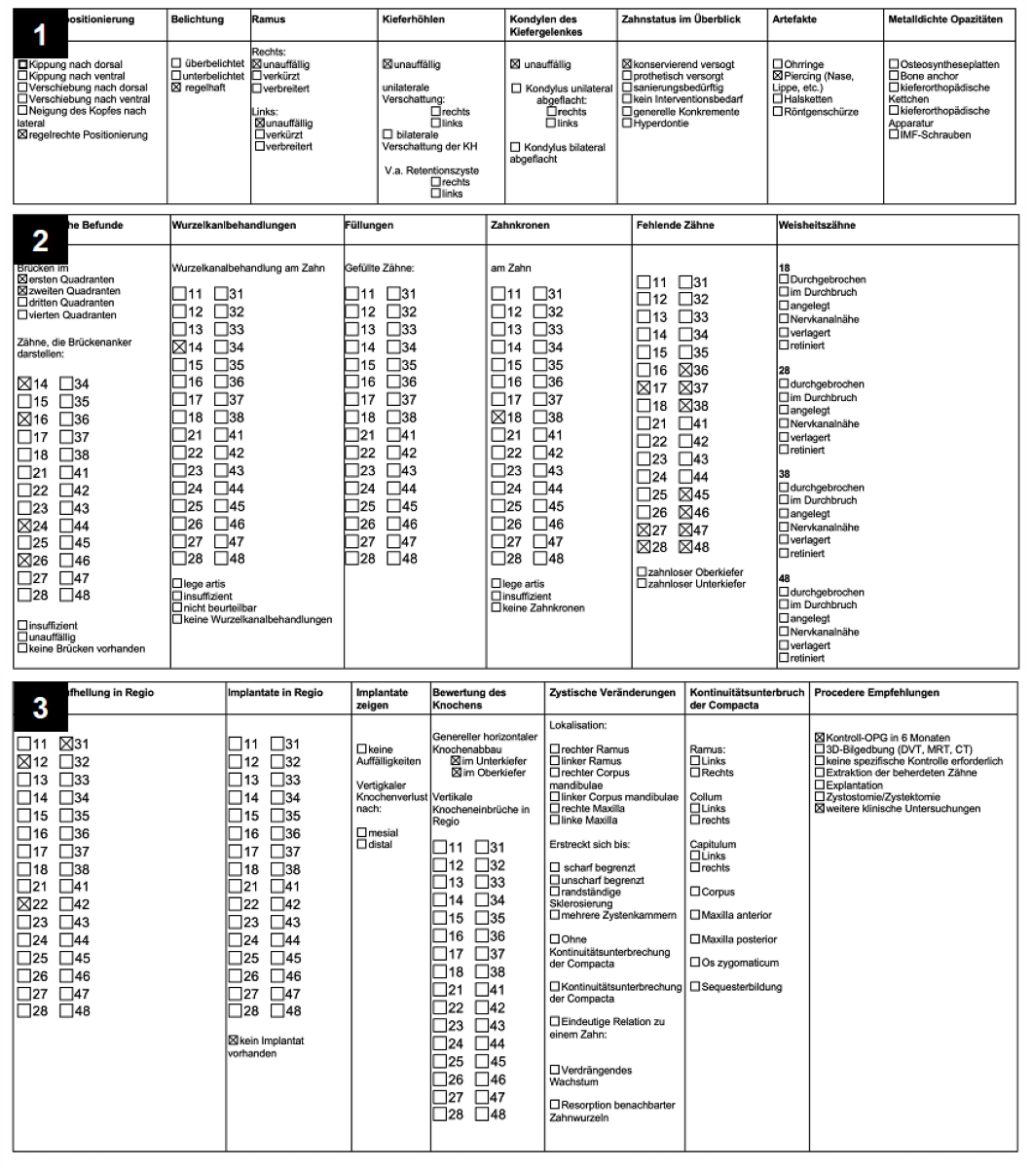
An example of a completed checkbox list containing 3 distinct spreadsheets (1, 2, and 3) used to generate radiology reports with ChatGPT.

Students were randomly assigned to 1 of 2 groups and presented with an unknown OPG (Figures 1A and 1B). Group A was instructed to analyze the x-ray image (Figure 1A) and compose a radiology report within 30 minutes without any external assistance. Group B received a second OPG (Figure 1B) and was tasked with completing a checkbox list (Figure 2) detailing their observations within a 10-minute time frame. These time limits were specifically chosen to investigate the potential time-saving benefits of using a structured checkbox method followed by AI-generated reporting. It was observed that all students used the entire allotted time for their respective tasks, neither exceeding the time limit nor completing early. The study, therefore, focused on the completion of the tasks within the predefined limits without measuring the exact duration for each task. Upon completion, each group was required to complete the alternate assignment with the opposite x-ray image. To minimize biases, the experimental design ensured that 1 group completed the checkbox for the same x-ray for which the other group composed the report, and vice versa. This approach reduced any influence of specific characteristics of the x-ray images (eg, the complexity of findings or difficulty of interpretation).

### Data Transformation and AI Text Generation

Upon completion, the checkbox lists filled out by participants were carefully transcribed into an Excel (Microsoft) data sheet comprising distinct spreadsheets for each category to organize the data. Subsequently, Chat GPT 4.0, an advanced AI language model, was harnessed to generate radiology reports based on the checkbox lists. Each spreadsheet within the Excel file was sequentially analyzed, and the information marked with an “X” in the “checkbox” column was incorporated into the generated reports. The following specific prompt was used to guide the AI in formulating structured x-ray reports, ensuring consistency and completeness:

Formulate a structured X-ray report in the sense of an X-ray report of an OPG based on the following checkbox list of the entire Excel table, and do not omit any columns. Please mention only those statements for which a box is marked with an X in the X-ray report. The statements not marked with an X should not be included in the report. The figures given should be interpreted in the sense of an odontogram. Analyze each spreadsheet in the Excel file in the order given. The column with the markings (X) is marked with the term “checkbox.” The report should be written in continuous text from the perspective of the treating dentist. Formulate a continuous text without subheadings.

The model settings included a temperature of 0.7 (controlling the randomness of responses), a maximum token limit of 1500 (restricting the length of the response), a frequency penalty of 0.0 (preventing repetitive word usage), and a presence penalty of 0.6 (promoting the inclusion of new topics). Those settings were shown to generate the highest output quality with the temperature setting of 0.7 being particularly important. Although lower settings are suggested to be advantageous for more deterministic tasks, preliminary tests revealed them to produce repetitive and difficult-to-read reports lacking naturalness and effectiveness in communication. In contrast, the chosen setting balanced creativity and coherence resulting in improved readability. Each report was generated using the ChatGPT web interface in a new session from September 5 to October 12, 2023, ensuring consistency and comparability across all outputs. To minimize biases associated with varying performance due to server load, which tends to be higher on weekends with higher traffic, the tasks were randomly distributed across different weekdays. This approach aimed to ensure a consistent and balanced evaluation of ChatGPT’s capabilities by reducing potential variability in output quality. The checkbox lists were directly uploaded without additional preprocessing.

### Readability Indices

The readability and complexity of both student-written and AI-generated texts were assessed using the Flesch reading ease (FRE) [24] score and the Lesbarhetsindex (LIX) readability index. “Readability” refers to how easily written material can be understood, determined by the complexity of the vocabulary, sentence, and word lengths used [23]. Although prior knowledge or motivation of the reader is not considered in readability formulas, especially in health care, a higher readability is associated with improved comprehension and participation of the patient.

The FRE score evaluates text readability based on its linguistic characteristics. In particular, the average sentence length (ASL) and the average number of syllables per word (ASW) are considered for the calculation using the following formula (adapted to the German language [25]):

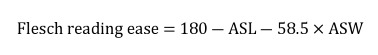



The FRE score typically ranges between 0 and 100, with higher scores indicating greater readability and lower scores suggesting increased complexity. Due to its high reproducibility [25], validation for various text types, and correlation with other readability formulas, the FRE score is an established metric in the analysis of medical texts [26-29].

LIX index considers the ASL and the prevalence of long words with more than 6 letters to assess text readability by the following calculation:

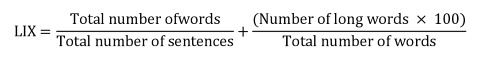



A higher LIX score indicates greater complexity, whereas a lower score suggests easier comprehension. LIX has been validated as a reliable measure of readability across multiple languages, including Swedish, Danish, English, French, German, Finnish, Italian, Spanish, and Portuguese [30,31].

To assess readability, the FRE and the LIX scores were calculated for both the student-written and AI-generated reports. Differences in readability were analyzed by comparing FRE and LIX scores of AI-generated reports with student-written reports. Additionally, this analysis was conducted collectively for all texts, as well as individually for each academic semester, to evaluate the influence of the educational level on text comprehensibility in comparison to automated text generation.

### Text Similarity (Bidirectional Encoder Representations from Transformers Score)

The accuracy of AI-generated texts was evaluated by comparing the number of findings diagnosed by students to those mentioned in the final AI-generated reports. Additionally, reference texts were manually created by a senior physician with extensive clinical experience in dental radiology, for each checkbox list to assess the quality of AI-generated texts. A comprehensive template was developed and carefully reviewed by all authors, serving as a standardized framework for report creation. Each reference text was individually crafted by transferring the findings from the corresponding checkbox list into the template. This standardized approach was consistently applied to each report, ensuring uniformity in content and structure while minimizing discrepancies that could bias the Bidirectional Encoder Representations from Transformers (BERT) score. These reference texts were then compared to the AI-generated text using the BERT score, a widely recognized metric for evaluating text similarity. Based on the BERT model [32], which generates high-dimensional vector representations (embeddings), capturing the BERT score measures the similarity between corresponding tokens in both texts. The BERT score includes 3 primary components—precision (P), recall (R), and *F*_1_-score. Precision measures the proportion of words in the AI-generated text that contribute accurately to the overall meaning as compared to the reference text. Essentially, it assesses the quality of the AI’s output in terms of the relevance and accuracy of the information presented. Recall evaluates the extent to which the AI-generated text covers all the relevant information contained in the reference text, highlighting how well the AI captures necessary details without omitting critical information. Finally, the *F*_1_-score provides a harmonic mean of precision and recall, offering a single score that balances both the completeness and accuracy of the AI-generated text. The aggregated similarity scores, normalized to a range between 0 and 1, indicate overall text similarity. A higher BERT score indicates textual similarity, reflecting a higher quality of AI-generated texts. Multiple studies have already demonstrated BERT’s capability of accurately predicting readability levels for various texts [33].

### Text Accuracy

The accuracy of AI-generated radiology reports was assessed by comparing the number of included findings with the number of findings contained in the referring checkbox list, both overall and for each of the 3 individual spreadsheets separately.

### Text Analysis

Descriptive text analysis was conducted by measurement of word count, sentence length, syllable count, diphthong count, and character count to compare AI-generated with student-written radiology reports. ASL and long word proportion (defined as words with more than 6 characters) were further assessed. Language quality across all texts was quantified by evaluating the error count including spelling, grammar, and punctuation together with the calculation of the error ratio (number of errors divided by words multiplied by 100). These metrics were analyzed collectively for all students and semesters as 1 group.

### Statistical Analysis

The software packages used for statistical analysis were GraphPad Prism 9.0 (Graphpad Software, LLC), G*Power 3.1 (Heinrich-Heine-University Düsseldorf), Excel 16.76, and SPSS Statistics (version 29; IBM Corp). To assess the potential difference in readability between AI-generated reports and those written by students, an a priori power analysis was performed. This analysis was based on previously observed significant differences in the FRE scores, which showed a lower average for ChatGPT responses (mean 34.9, SD 11.2) compared to medical information on Google webpages (mean 46.5, SD 14.3), accounting for a difference of 11.6 [34]. Additionally, similar results with the LIX score have demonstrated a difference of 10 between human-written and ChatGPT-written scientific introductions [35]. To achieve a power of 80% and maintain a significance level of 5%, a minimum of 25 samples per group (study arm) is required. Significance was set at *P*<.05. All data are presented as mean (SD). Differences between student-written and AI-generated texts were analyzed using a 2-tailed student *t* test. A subsequent post hoc power analysis was conducted for each test to verify the power achieved by the *t* test.

## Results

### Overview

Text quality, readability, and comprehensibility of student-written and AI-generated radiology reports were compared by analysis of various language parameters. Throughout the study, students consistently used the preset time to its full extent, dedicating 30 minutes for completing the written report and 10 minutes for the competition of the checkbox list. While AI-generated radiology reports demonstrated a remarkable similarity to reference texts with no difference in readability, a significant information deficiency was observed.

### AI-Generated and Student-Written Texts Possessed Identical Readability

**Figure 3:**
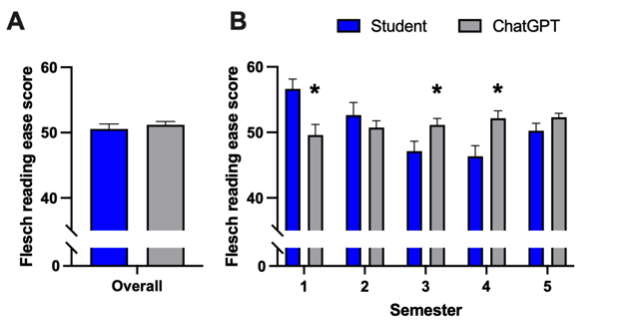
Metric evaluation of readability of AI-generated reports compared to student-written radiology reports (A) overall and (B) individually for each semester assessed with the Flesch readability ease score. Data represent mean (SD). Sample size: (A) n=100; (B) semester 1: n=20, semester 2: n=19, semester 3: n=21, semester 4: n=20, and semester 5: n=20; *P<.05; and versus students. AI: artificial intelligence.

The FRE score (Figure 3A,B) revealed no difference in readability between AI-generated and student-written texts (mean 50.55, SD 7.80 vs mean 51.19, SD 5.02) considering all reports together as demonstrated in Figure 3A (*P*=.49; t_198_=0.6898). Upon examination of each semester individually, the Flesch index exhibited significant variability, with AI-generated texts demonstrating lower readability compared to texts written by the first clinical semester (mean 56.65, SD 6.70 vs mean 49.6, SD 7.17; *P*=.002; t_38_=3.213) and higher readability compared to the third (mean 47.14, SD 6.97 vs mean 51.14, SD 4.55; *P*=.03; t_40_=2.203) and fourth (mean 46.35, SD 7.29 vs mean 52.15, SD 5.08; *P*=.006; t_38_=2.918) clinical semesters. No difference was found for second (*P*=.39; t_36_=0.8647) and fifth (*P*=.12; t_38_=1.577) clinical semester.

**Figure 4:**
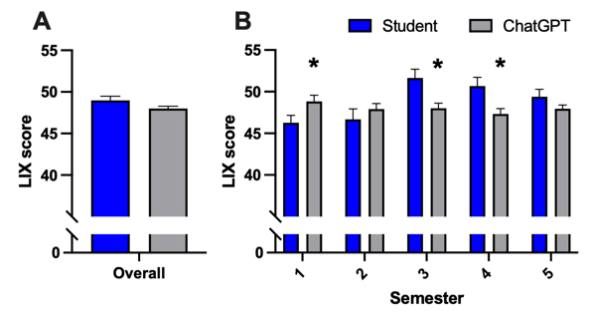
Metric evaluation of readability of AI-generated reports compared to student-written radiology reports (A) overall and (B) individually for each semester assessed with LIX index. Data represent mean (SD). (A) Sample size: n=100; (B) semester 1: n=20, semester 2: n=19, semester 3: n=21, semester 4: n=20, and semester 5: n=20; *P<.05; and versus students. AI: artificial intelligence; LIX: Lesbarhetsindex.

As presented in Figure 4A, no overall difference between both groups regarding readability was found (mean 48.98, SD 5.0 vs mean 48.0, SD 2.85) as assessed with the LIX index (*P*=.09; t_198_=1.699). In contrast to the FRE score, the LIX readability index exhibited opposing trends across semesters (Figure 4B), with significant differences observed in semesters 1 (mean 46.27, SD 4.0 vs mean 48.81, SD 3.44; *P*=.04; t_38_=2.157); 3 (mean 51.64, SD 4.89 vs mean 48.01, SD 2.84; *P*=.005; t_40_=2.944); and 4 (mean 50.67, SD 4.68 vs mean 47.32, SD 2.90; *P*=.098; t_38_=2.719). No difference was observed in the second (*P*=.39; t_36_=0.877) and fifth (*P*=.15; t_38_=1.464) clinical semester.

### AI-Generated Reports Show Great Similarity to Reference Texts but Lack Information

**Figure 5:**
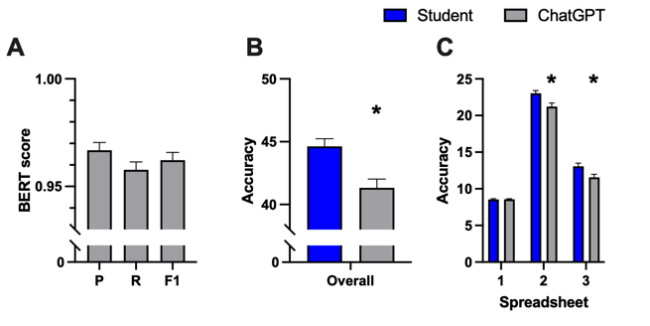
Evaluation of similarity compared to reference texts using the (A) BERT score with precision (P), recall (R), and F1 score (F1) representing the harmonic mean of precision and recall. The accuracy of AI-generated radiology reports was further assessed as (B) overall accuracy including the whole checkbox list and (C) individually for each spreadsheet of the checkbox list. Data represent mean (SD). Sample size: n=100; *P<.05 versus students. AI: artificial intelligence; BERT: Bidirectional Encoder Representations from Transformers.

As illustrated in Figure 5A, the great similarity is indicated by a high BERT score, with precision (P)=mean 0.967, SD 0.036, recall (R)=mean 0.958, SD 0.037, and *F*_1_=mean 0.962, SD 0.036. The analysis further revealed a notable deficiency in relevant information within AI-generated texts. A significant difference was evident between the findings diagnosed by students and those mentioned in the AI-generated reports (Figure 5B), with students identifying a mean of 44.6 (SD 6.0) findings, whereas the AI reported a mean of 41.3 (SD 7.0) findings in total (*P*=.04; t_198_=3.586). Specifically, as shown in Figure 5C, while no difference was observed in the first spreadsheet (mean 8.53, SD 1.06 vs mean 8.55, SD 1.02; *P*=.89; t_198_=0.1361), the AI included significantly fewer findings from the second (mean 23.03, SD 3.67 vs mean 21.22, SD 4.92; *P*=.003; t_198_=2.951) and third (mean 13.07, SD 4.40 vs mean 11.56, SD 4.23; *P*=.014; t_198_=2.476) spreadsheets.

### AI-Generated Significantly Shorter and Error-Free Radiology Reports

**Figure 6:**
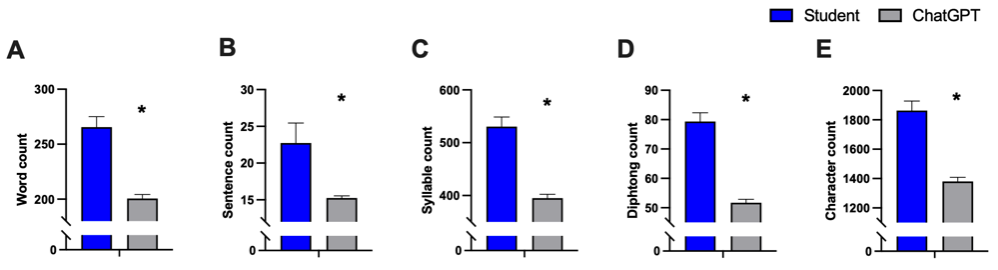
Analysis of (A) word count, (B) sentence count, (C) syllable count, (D) diphthong count, and (E) character count of AI-generated radiology reports compared to student-written reports. Data represent mean (SD). Sample size: n=100; *P<.05 versus students. AI: artificial intelligence.

AI-generated radiology reports exhibited a significant 24% reduction in word count (mean 265.6, SD 95.4 vs mean 200.6, SD 37.3 words; *P*<.01; t_198_=6.347) and sentence count (*P*=.007; t_198_=2.726) accompanied by significant reductions in syllables (*P*<.01; t_198_=6.823), diphthongs (*P*<.01; t_198_=8.643), and characters (*P*<.01; t_198_=6.841) compared to student-written texts as presented in Figure 6.

**Figure 7:**
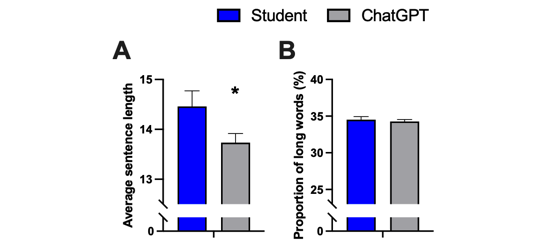
Analysis of sentence length and long word proportion (more than 6 characters) of AI-generated radiology reports compared to student-written reports. Data represent mean (SD). Sample size: n=100; *P<.05 versus students. AI: artificial intelligence.

Whereas radiology reports generated by AI showed a significant reduction in ASL compared to student-written reports (A: mean 14.5, SD 3.1 vs mean 13.7, SD 1.8 words; *P*=.046; t_198_=2.007), no difference was observed regarding the proportion of long words (B: *P*=.61; t_198_=0.509) and this is presented in Figure 7.

**Figure 8:**
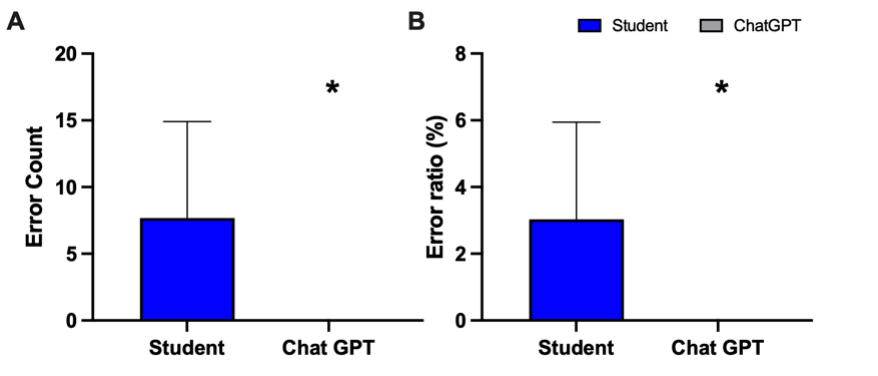
Analysis of (A) error count including grammar, spelling, and punctuation and (B) error ratio by calculating errors divided by words multiplied by 100 of student-written radiology reports and AI-generated radiology reports. Data represent mean (SD). Sample size: n=100; *P<.05 versus students. AI: artificial intelligence.

Contrary to student-written reports, AI-generated texts showed a complete absence of orthographic, grammatical, and punctuation errors as presented in Figures 8A and 8B (A: mean 7.7, SD 7.2 vs mean 0; *P*<.01; t_198_=10.59; B: mean 2.9, SD 2.9 vs mean 0; *P*<.01; t_198_=10.41).

### Student-Written Reports Showed Significant Differences in Length and Readability Across Semesters

**Figure 9:**
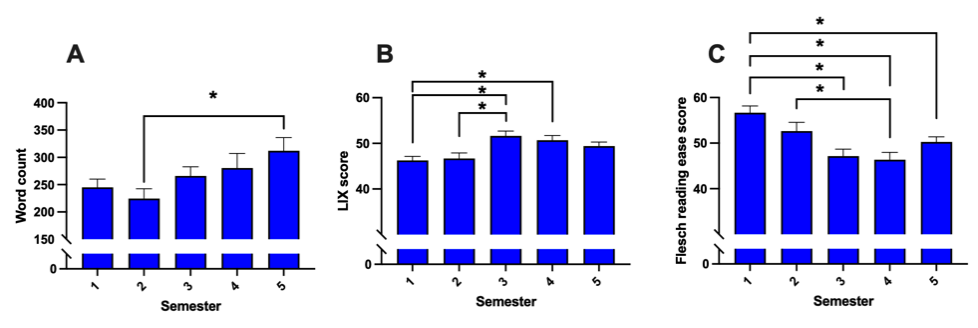
(A) Analysis of word count, (B) metric evaluation of readability using LIX score, and (C) Flesch reading ease score of student-written radiology reports. Data represent mean (SD). Sample size: n=100; semester 1: n=20, semester 2: n=19, semester 3: n=21, semester 4: n=20, and semester 5: n=20. *P<.05. LIX: Lesbarhetsindex.

Student-written reports showed a significant difference in word count across different semesters (*P*=.04; t_99_=1.610), with a noticeable trend toward the use of more words in higher semesters (Figure 9A; semester 2 vs semester 5: mean 224, SD 79 vs mean 312, SD 107; *P*=.03; t_95_=2.97). Additionally, significant differences in readability were observed across semesters, as presented in Figures 9B and 9C (LIX score: *P*=.06; t_99_=2.315; FRE score: *P*<.01; t_99_=2.762). Students from semesters 1 and 2 produced simpler and easier-to-understand reports, whereas those from higher semesters tended to write more complex and, hence, more difficult-to-read reports.

## Discussion

### Principal Findings

Integrating AI into clinical workflows and medical education has attracted significant interest due to its potential to enhance efficiency. Our study, therefore, aimed to investigate the effectiveness of AI-generated radiology reports by comparing them to student-written reports. In summary, no difference regarding readability was found between the AI-generated and student-written radiology reports. Whereas AI-generated reports showed an overall high textual similarity to reference texts, they simultaneously lacked substantial diagnostic information. Noteworthy, the language quality was significantly improved compared to student-written reports, with AI-generated texts being completely error-free. The results revealed high potential in enhancing medical writing with AI while still being limited by the reliability of the transferred information [36].

AI is revolutionizing medicine across diagnosis, treatment, and administrative tasks [5]. AI algorithms analyze medical images for early disease detection, provide clinical decision support, and enable personalized treatment plans [3]. In drug development, AI accelerates processes by predicting drug interactions and screening compounds [2]. Whereas the capability of AI to diagnose x-ray images has already been proven [4], the process of diagnosing was excluded in this study to focus on generating radiology reports based on information collected by students.

Indicated by an overall high BERT score, our findings prove that AI-generated radiology reports exhibit a great level of similarity to reference texts. Integration of ChatGPT into the medical documentation process, therefore, results in high text quality as represented by high precision, recall, and *F*_1_-score, which further highlights the overall robustness of AI in replicating the content of reference reports only based on checkbox information. Concomitantly, after the preparation of preliminaries regarding the prompt and template design, automating the process of report writing with ChatGPT results in significant time savings and, hence, highlights the potential to streamline the workflow for health care professionals. Although this study did not aim to specifically quantify time efficiency, AI-supported report generation was noticeably quicker due to the shorter time cap (10 minutes for checkbox list completion vs 30 minutes for report writing). Whereas all students were observed to use the entire allotted time, likely focusing on thoroughness and ensuring report completeness, rather than being constrained by the time limit, future research could incorporate exact time measurements and posttask surveys to gather participant feedback on time allocation to provide additional insights regarding the adequacy of the time frames. The successful use of ChatGPT in composing medical notes related to patient transfers, operative procedures, and surgical assistance [5,18,37] underscores its role in enhancing productivity within medical environments, thereby highlighting the transformative impact of AI-driven technologies in health care.

Prior to the study, the prompt design was refined extensively, with multiple versions tested to optimize the AI’s output. Ultimately, only the most effective prompt was selected to continue generating reports, ensuring the highest possible accuracy in AI-generated text. However, despite their overall similarity, AI-generated reports demonstrated a significant deficiency in relevant information, indicating a crucial impact of the prompt provided to ChatGPT in determining the accuracy of the results. This discrepancy was particularly evident in identifying and incorporating findings regarding specific teeth, with AI-generated reports containing significantly fewer findings compared to the number of diagnoses documented with the checkbox lists (eg, AI-generated reports did not mention the presence of a cyst, the status of dental restorations or precise prescription of bone loss). Interestingly, while no difference was observed in the findings reported from the first spreadsheet, a significant disparity emerged in subsequent spreadsheets. The potential limitation in the AI’s ability to comprehensively interpret complex odontogram data leads to inconsistencies in the inclusion of relevant findings. These findings underscore the error-prone interplay between prompt precision, image complexity, and AI performance in radiology reporting.

In contrast to missing information, another known challenge in the use of ChatGPT is its tendency to generate plausible-sounding but incorrect or fabricated information, commonly referred to as “hallucinations” [38]. However, this study showed no indication that ChatGPT included invented findings not present in the original checkbox list, as evidenced by the high BERT score. The prompt design strictly instructed the AI to use only information from the checkbox list, thereby minimizing the risk of hallucinations. Our observations confirmed that the model adhered to these guidelines. Although the prompt instructed ChatGPT to interpret the numbers as odontogram information to identify each tooth, we encountered challenges in consistently incorporating and accurately understanding the provided data. Precision in prompt formulation emerges as a critical factor influencing the accuracy and completeness of AI-generated reports [39]. The formulation of prompts significantly influences the outcomes generated by AI systems, with precise prompts being necessary to provide clear instructions and context for the AI model, guiding it in producing relevant and accurate responses. The specificity and clarity of the prompt directly impact the quality and relevance of the AI-generated output [40]. The design of effective prompts, therefore, remains a crucial part of future research.

Regarding radiology reports, a prompt that precisely outlines the required structure, format, and content of the report will likely result in more coherent outputs. Moreover, the prompt helps the AI model understand the task and focus on relevant information. By providing detailed guidelines and constraints, the prompt narrows the scope of the AI’s search and directs it toward generating responses that align with the desired objectives. Additionally, prompts can incorporate domain-specific terminology and concepts to ensure that the AI model produces contextually appropriate and clinically relevant outputs. Nonetheless, a potential bias of machine learning systems must be considered due to their susceptibility to being influenced by the training data, thereby generating biased or misleading outputs. Well-designed prompts can help mitigate these issues by guiding the AI model toward more objective and accurate responses [41]. Moreover, the challenges associated with interpreting complex diagnostic data like orthopantomograms emphasize the need for continuous refinement and optimization of AI algorithms to ensure reliable performance in a clinical setting. Addressing these challenges will require a collaborative effort between clinicians, AI developers, and educators. Enhancing prompt precision through detailed guidelines and standardized protocols can improve AI performance and reduce information deficiencies in generated reports. Notably, to realize the full potential of AI in health care, the risk of disseminating misinformation must be mitigated. The rapid spread of false or misleading content, commonly called infodemic, highlights the importance of implementing validation mechanisms to ensure the reliability of AI-generated content [42,43].

In the context of this study, the AI was not supposed to formulate diagnoses independently but rather to generate radiology reports based explicitly on the findings and diagnoses provided by the students. This approach evaluated the AI’s ability to effectively translate diagnostic information into coherent and comprehensive reports, reflecting real-world clinical scenarios of radiologists interpreting images and automatically converting their findings into written reports. Overall, this evaluation of AI-generated reports underscores the reliability and consistency of AI in producing error-free content compared to student-written texts. Remarkably, AI-generated reports exhibited a considerable reduction in word count, sentence count, and various linguistic features, including syllables and diphthongs. This reduction in length was accompanied by a notable absence of orthographic, grammatical, and punctuation errors, highlighting the accuracy and precision of AI-generated text. Moreover, no discernible difference between AI-generated and student-written radiology reports was observed regarding their readability. Both sets of reports demonstrated similar readability levels as indicated by the FRE score and LIX index, with both being established and validated as reliable measures for assessing text difficulty, including medical texts [27,29,30]. However, examination of individual semesters revealed significantly lower readability for AI-generated reports than student-written ones in the first clinical semester, but higher readability compared to the third and fourth clinical semesters. A possible explanation could be the use of more advanced and specialized terminology by the AI compared to students in the first semester, resulting in lower readability scores. Reports from students in the first semester may adhere to a simpler structure, reducing difficulty and increasing readability and comprehension. In contrast, reports from the third and fourth semesters exhibit more complexity in structure and terminology to present diagnostic information due to extensive expertise and, therefore, impairing readability. These findings are supported by the significant differences in word count and readability across all semesters upon individual examination. Students in lower semesters tend to use fewer words and write reports with higher readability, whereas students from higher semesters tend to write longer, more complex, and therefore, more difficult-to-read reports. As students progress through their education, their increased clinical experience and familiarity with radiological terminology likely enhance the quality of their reports. This development is reflected by the incorporation of advanced terminology and structure, indicating a clear learning curve. The variability in skill development across semesters significantly impacts the comparison between student-written and AI-generated reports, potentially affecting the comparison in favor of later semesters. This disparity underscores the importance of considering skill levels when evaluating AI performance since differences in student proficiency could lead to variability in report quality, affecting readability and accuracy metrics. Consequently, the perceived quality of AI-generated reports may vary depending on the student cohort they are compared with, highlighting the necessity of accounting for student skill differences in the study design and analysis. However, on the other hand, the variability in student skills across semesters positively reflects the diverse real-world conditions in clinical practice, where practitioners exhibit a range of expertise. This diversity in the study cohort allows the AI-generated reports to be tested against various levels of proficiency, demonstrating the AI’s potential to support users with different levels. Early-stage dental students could benefit from a structured and consistent framework provided by AI, enhancing their learning and understanding. Advanced students and experienced clinicians could use AI to reduce repetitive tasks and ensure accuracy in documentation, allowing more focus on diagnostic decision-making. AI assistance in diagnostics has been further shown to improve performance, though radiologists often underweight AI predictions [44]. Overall, AI tools can support a wide range of users by adapting to their specific needs and improving educational and clinical outcomes. This aligns with study results proving GPT-4 to enhance productivity and quality in various tasks beyond medical use, benefiting consultants and customer support agents across all skill levels [45,46].

Nevertheless, the differentiated use of reports must be considered due to the diverse communication needs within health care settings. On the one hand, health care professionals require detailed reports for accurate clinical decision-making and effective interprofessional communication. On the other hand, patients benefit from simpler, more understandable reports to understand their medical conditions and actively engage in treatment discussions. AI has been further shown to efficiently simplify medical data for better patient understanding [22]. Hence, its implementation offers the possibility to fulfill both the detailed requirements of health care professionals and the simplified needs of patients simultaneously in response to 2 different prompts. Consequently, automated AI solutions could facilitate effective communication among health care providers and increase patient empowerment and participation beyond time-saving and more efficient documentation in health care.

### Conclusions

In conclusion, AI’s potential to enhance medical writing efficiency is highlighted, yet remaining challenges in ensuring reliability and comprehensiveness must be faced. The precision of prompts significantly impacts AI’s accuracy, particularly in interpreting complex diagnostic data. Future research should focus on refining AI algorithms and prompt design to optimize medical reporting. Overall, integrating AI-driven solutions into routine clinical workflows offers a practical tool for enhancing productivity.

## Abbreviations

AI: artificial intelligence
ASL: average sentence length
ASW: average number of syllables per word
BERT: Bidirectional Encoder Representations from Transformers
FRE: Flesch reading ease
OPG: panoramic radiograph
